# Effect of comprehensive nursing intervention on the efficacy of spleen aminopeptide combined with aerosol inhalation in the treatment of pediatric pneumonia

**DOI:** 10.12669/pjms.39.4.7195

**Published:** 2023

**Authors:** Yuan Yue, Qian Liang, Lei Shi, Wen-jing Bai, Juan Fu

**Affiliations:** 1 Yuan Yue Department of Outpatient, Baoding Children’s Hospital, Baoding 071000, Hebei, China; 2 Qian Liang Department of Emergency, Baoding Children’s Hospital, Baoding 071000, Hebei, China; 3 Lei Shi, Department of Outpatient, Baoding Children’s Hospital, Baoding 071000, Hebei, China; 4 Wen-jing Bai Department of Outpatient, Baoding Children’s Hospital, Baoding 071000, Hebei, China; 5 Juan Fu Department of Outpatient, Baoding Children’s Hospital, Baoding 071000, Hebei, China

**Keywords:** Pediatric Pneumonia, Comprehensive Nursing, Aerosol Inhalation, Efficacy

## Abstract

**Objective:**

To analyze the effect of comprehensive nursing intervention on the efficacy of spleen aminopeptide combined with aerosol inhalation in the treatment of pediatric pneumonia.

**Methods:**

This is a retrospective study. Eighty children with pneumonia admitted to Baoding children’s Hospital from March 2020 to March 2021 were included and randomly divided into two groups. Children in the control group received routine treatment and nursing measures, while those in the experimental group received comprehensive nursing intervention on the basis of routine treatment in the control group. The differences in clinical effect, symptom improvement time, nursing quality score and satisfaction score between the two groups were compared and analyzed.

**Results:**

The efficacy of the experimental group was significantly higher than that of the control group (p=0.02). After comprehensive nursing intervention, the cough disappearance time, body temperature recovery time, pulmonary rales disappearance time and hospitalization time in the experimental group were significantly shorter than those in the control group, with statistically significant differences (p<0.05). The scores of nursing quality such as health guidance, nursing operation, and medication management in the experimental group were higher than those in the control group, with significant differences in the data comparison between the groups (p<0.05). The satisfaction of the experimental group was 100%, which was higher than 90% of the control group, with a statistically significant difference (p=0.04).

**Conclusion:**

Comprehensive nursing intervention boasts various significant effects in the treatment of pediatric pneumonia, such as rapid amelioration of the condition, improvement of efficacy, and enhancement of nursing quality and satisfaction.

## INTRODUCTION

Pediatric pneumonia is a pulmonary inflammation caused by viral or bacterial infection, which is one of the common and frequent-occurring diseases in pediatrics. If children suffering from this disease cannot be treated with appropriate treatment measures in a timely manner, not only their physical and mental health will be seriously affected, but even their lives will be threatened.^1^ According to clinical practice, children with pneumonia recover slowly if given simple anti-inflammatory and antiviral therapy, and are easy to be complicated with other severe complications such as myocarditis.^2^

In contrast, aerosol inhalation therapy can effectively relieve the clinical symptoms of pediatric pneumonia. Spleen aminopeptide combined with aerosol therapy boasts a significant improvement in the therapeutic effect of pediatric pneumonia. For modern medical clinical care, a series of nursing interventions should be implemented to achieve the effective purpose of improving the treatment compliance and treatment effect of children.

Comprehensive nursing intervention is a new type of nursing method, which is to provide nursing care in a predetermined method based on the condition of the child for a certain disease.^3^ A growing body of evidence^4^ suggests that comprehensive nursing improves child-related quality of life and reduces health service use. However, to date, no application of this regimen in pediatric pneumonia has been reported. In this study, comprehensive nursing intervention was used to investigate the clinical effect of splenic aminopeptide combined with aerosol inhalation in the treatment of pediatric pneumonia, and a certain effect was achieved.

## METHODS

This is a retrospective study. Eighty children with pneumonia admitted to Baoding Children’s hospital from March 2020 to March 2021 were recruited and randomly divided into two groups: the control group and the experimental group, with 40 cases in each group. No significant difference was observed in the comparison of general data between the two groups, which was comparable (Table-I).

**Table-I:** Comparative analysis of the general data of the experimental group and the control group (*χ̅*±*S*) n=40.

Index	Experimental group	Control group	t/χ^2^	P
Age (years)	6.32±4.51	6.27±4.23	0.05	0.96
Male (cases %)	22	24	0.20	0.65
Course of disease (days)	8.61±1.32	8.34±1.06	1.02	0.32
Type of pneumonia				
Bacterial pneumonia	18	20	0.23	0.61
Viral pneumonia	14	15	0.05	0.82
Other types of pneumonia	8	5	0.83	0.36

p>0.05.

### Ethical Approval:

The study was approved by the Institutional Ethics Committee of Baoding children’s Hospital (No.:2022024; date: June 18, 2021), and written informed consent was obtained from all participants.

### Inclusion criteria:

- Patients who meet the criteria for diagnosis and treatment of pneumonia^5^;
- Patients with clear lung lesions that can be accurately measured by imaging examination;
- Patients aged ≤12 years old;
- Patients with informed consent from family members;
- Patients with no allergies or contraindications to the applied drugs;
- Patients with complete clinical data.

### Exclusion criteria:

- Patients with severe systemic diseases;
- Patients with severe organ function injury or failure;
- Patients with coagulopathy;
- Patients with severe congenital diseases.

Children in the control group were given routine treatment and nursing measures, including cough relief, oxygen inhalation, maintenance of body water, electrolytes, pH balance and other rehydration, antiviral and antibiotic treatment, aerosol inhalation to dispel phlegm and relieve asthma, and oral administration of spleen aminopeptide lyophilized to enhance immunity. The specific usage of spleen aminopeptide oral lyophilized powder is as follows: 2-mg/time, once daily. Medication and symptomatic treatment with routine nursing measures were given, such as regular temperature measurement and observation of changes in vital signs.

Those in the experimental group received comprehensive nursing intervention on the basis of routine treatment in the control group:

### Environmental intervention:

Improving the surrounding environment conditions based on the children’s own conditions, creating a warm and comfortable environment, opening windows for ventilation, and adjusting indoor temperature and humidity according to indoor and outdoor temperature changes to improve comfort. In addition, regular disinfection was carried out under ultraviolet light to prevent cross-infection.

### Health guidance:

Health animation was used to promote health education for children. Diversified means, such as the distribution of health manuals, the transmission of knowledge on posters on hospital bulletin boards, and the playback of micro-educational videos, have imprinted the knowledge of pediatric pneumonia in the hearts of parents, thereby increasing their awareness of disease knowledge.

### Cough intervention:

After sputum removal, nursing personnel adjusted the nursing services according to the severity of children’s illness, and gave cough medication to those with more severe cough. If there is shortness of breath after cough, children should be given oxygen inhalation therapy in time.

### Psychological intervention:

Nursing personnel frequently contacted the children and shortened the relationship with them according to the actual situation of the children, in order to alleviate their fear and improve the degree of cooperation. The children had their body temperature checked daily.

### Dietary intervention:

The children ate three meals a day with nutritious, less greasy food and more fresh fruits and vegetables.

### Exercise:

After the condition improved, parents were instructed to let the children exercise a little more frequently. The maximum follow-up time for patients in both groups was six months. And case data collection ceased in March 2021.

### Evaluation of clinical efficacy one week after treatment:

***Markedly effective:*** Symptoms basically disappeared, and imaging examination showed that the children’s pulmonary inflammation was completely absorbed. ***Effective:*** Symptoms significantly disappeared, and pulmonary inflammation was partially absorbed. ***Ineffective:*** No improvement in symptoms, the pulmonary inflammation was not absorbed and even aggravated. Effective rate = (markedly effective + effective)/total number of cases × 100%.^6^ Symptom improvement time: The cough disappearance time, pulmonary rales disappearance time, body temperature recovery time and hospitalization time after nursing were recorded and counted. Comparative analysis of nursing quality score: including health guidance, nursing operation, medication management. The score ranges from 0 to 100, with higher scores indicating higher nursing quality.

### Comparative analysis of satisfaction:

The Patient Satisfaction Questionnaire Short Form (PSQ-18)^7^ was used to investigate the satisfaction of family members of two groups of children with pneumonia. The total score is 100 points, with 80 points or more being very satisfied, 60-80 points being satisfied, and less than 60 points being dissatisfied. Satisfaction rate = (number of satisfied cases + number of basic satisfied cases)/total number of cases × 100%.

### Statistical Analysis:

All data in this study were statistically analyzed by SPSS 20.0 software, and measurement data were expressed as (*χ̅*±*S*). Two independent sample t-test was used for comparison between groups, paired t test was used to analyze data within groups, and c^2^ test was used for the comparison of rates. P<0.05 indicates a statistically significant difference.

## RESULTS

The comparative analysis of the clinical efficacy between the two groups showed that the efficacy of the experimental group was 97.5%, which was significantly higher than 82.5% of the control group (p=0.02) (Table-II).

**Table-II:** Comparative analysis of the clinical efficacy of the two groups (*χ̅*±*S*) n=40.

Group	Markedly effective	Effective	Ineffective	Total effective rate
Experimental group	33	6	1	39 (97.5%)
Control group	25	8	7	33 (82.5%)
*c* ^2^				4.74
P				0.02

p<0.05.

After comprehensive nursing intervention, the cough disappearance time, body temperature recovery time, pulmonary rales disappearance time and hospitalization time in the experimental group were significantly shorter than those in the control group (p<0.05) (Table-III). The scores of nursing quality such as health guidance, nursing operation, and medication management in the experimental group were higher than those in the control group (p<0.05) (Table-IV). The satisfaction of the experimental group was 100%, which was higher than 90% of the control group (p=0.04) (Table-V).

**Table-III:** Comparative analysis of symptom improvement time before and after intervention in the two groups (*χ̅*±*S*) n=40.

Group	Cough disappearance	Body temperature recovery	Hospitalization time	Pulmonary rales disappearance
Experimental group	4.33±1.20	5.03±2.04	8.23±2.16	5.76±1.34
Control group	5.27±1.46	6.31±2.17	9.57±2.39	6.67±1.40
t	3.15	2.72	2.63	2.97
P	0.00	0.01	0.01	0.00

p<0.05.

**Table-IV:** Comparative analysis of nursing quality scores between the two groups (*χ̅*±*S*) n=40.

Group	Health guidance	Nursing operation	Medication management
Experimental group	96.55±7.34	95.84±5.72	96.76±6.48
Control group	85.76±8.03	83.21±6.41	87.90±8.42
t	6.27	9.30	5.27
p	0.00	0.00	0.00

*p<0.05.

**Table-V:** Comparative analysis of satisfaction between two groups (*χ̅*±*S*) n=40.

Group	Very satisfied	Satisfied	Not satisfied	Total satisfaction*
Experimental group	35	5	0	40 (100%)
Control group	33	3	4	36 (90%)
*c* ^2^				4.21
P				0.04

* p<0.05

## DISCUSSION

Comprehensive nursing intervention is a commonly used nursing method in clinical practice, which can be determined by combining the situation of each patient, nursing outcomes and the functional rehabilitation potential of patients.^8^ It was found in our study that the efficacy after intervention in the experimental group was higher than the control group. After comprehensive nursing intervention, the cough disappearance time, body temperature recovery time, pulmonary rales disappearance time and hospitalization time in the experimental group were significantly shorter than those in the control group.

It can be seen that comprehensive nursing intervention in pediatric pneumonia is helpful to shorten the symptom improvement time and facilitate the recovery of children. Roesler et al.^9^ Considered that the comprehensive nursing intervention model could apply the latest nursing methods and nursing measures to clinical practice and provide patients with continuous and high-quality rehabilitation nursing. In addition, the implementation of comprehensive nursing intervention is beneficial to improve the quality of life of patients, improve their health behaviors, help to develop good living habits, and avoid bad lifestyles.^10^

Pneumonia is a common infectious disease of upper respiratory tract in children. Despite major advances in its prevention and treatment, pneumonia remains the primary cause of death in children outside the neonatal period.^11^ There are many risk factors leading to pediatric pneumonia, including age, malnutrition, immunosuppression, pathogenic microbial infection, etc.,^12^,^13^ among which pulmonary inflammation caused by pathogens such as viruses and bacteria is the most common.^14^ Pediatric pneumonia is mainly manifested clinically as fever, cough, shortness of breath and dyspnea, wet rales on lung auscultation, and inflammatory lesions found on chest imaging examination.

For pediatric pneumonia, the main treatment measures can be divided into etiological treatment and symptomatic treatment. When taking etiological treatment, antiviral vitamin therapy is the preferred method.^15^ Symptomatic treatment is not inferior to etiological treatment in clinical practice, boasting various benefits such as keep the children’s respiratory tract patency, instructing children to effectively cough and suck sputum, correcting children’s hypoxia. Budesonide aerosol inhalation can directly act on the pharynx and trachea of children with pneumonia, boasting significant relief of hypoxia in children, rapid increase of oxygen concentration, obvious clinical effect.^16^ Spleen aminopeptide oral lyophilized powder is mainly composed of polypeptides and nucleotides extracted from fresh pig spleen, which contain a variety of amino acids and immunomodulatory factors. Clinically, it is widely used in the treatment of cellular immune deficiency, immune deficiency or other infections due to its obvious therapeutic advantages for infectious diseases, which can trigger and enhance the cellular immune function of the body, and promote the immune balance of the body.^17^

Due to the young age of children and the influence of diseases, some children are unable to accurately cooperate with drug treatment. Comprehensive nursing is centered on children, emphasizing the continuous improvement of the quality of nursing work through a variety of nursing measures, providing more comprehensive, systematic and targeted nursing measures for children. Pediatric comprehensive nursing plays an important role in crisis intervention; However, few studies have investigated the effect of comprehensive nursing in the treatment of pediatric pneumonia.^18^ Comprehensive nursing has important advantages in children’s mental health comprehensive care and behavioral health care.^19^ Cooper et al. held that while actively preventing infection and complications, comprehensive nursing intervention could accelerate the recovery of children and reduce the incidence of complications.^20^

In this study, the scores of nursing quality such as health guidance, nursing operation, and medication management in the experimental group are higher than those in the control group. The satisfaction of the experimental group was higher than that of the control group. The conclusion of this study provides a new reference for the application of comprehensive nursing intervention in the treatment of pediatric pneumonia.

### Limitations:

It includes a small sample size with a short follow-up period. In response to this, more samples will be included in future clinical studies and follow-up time will be extended, in order to more objectively evaluate the advantages and disadvantages of comprehensive nursing intervention and benefit more patients.

## CONCLUSION

Comprehensive nursing intervention boasts various significant effects in the treatment of pediatric pneumonia, such as rapid amelioration of the condition, improvement of efficacy, and enhancement of nursing quality and satisfaction.

### Authors’ Contributions:

**YY and QL:** Carried out the studies, participated in collecting data, drafted the manuscript, are responsible and accountable for the accuracy and integrity of the work.

**LS:** Performed the statistical analysis and participated in its design.

**WB:** Participated in acquisition, analysis, or interpretation of data and draft the manuscript.

All authors read and approved the final manuscript.
